# Notes on Dermatology

**Published:** 1894-08

**Authors:** Isadore Dyer

**Affiliations:** New Orleans, La., Professor of Dermatology in the New Orleans Polyclinic; Lecturer and Clinical Instructor in Skin Diseases, Medical Department Tulane University, Etc.


### Current Medical Literature.

### NOTES ON DERMATOLOGY.

BY ISADORE DYER, M. D., NEW ORLEANS, LA.,
Professor of Dermatology in the New Orleans Polyclinic; Lecturer and Clinical Instructor in Skin Diseases, Medical Department Tulane University, etc.

THE PRESENT POSITION OF THE LICHEN QUESTION.—In 1889, at the Paris Dermatological Congress, Kaposi reduced the lichen group to two diseases: 1. Lichen ruber; a, acuminatus; b, planus. 2. Lichen scrofulosorum.

Since that congress, comparatively little has appeared in the journals upon the subject of lichen.

Brocq and Jacquet say that the basis for the construction of the lichen group is to be found in the presence of the initial lesion of papular character. In summarizing a very interesting paper on this timely subject, Dr. Malcolm Morris submits his own conclusions as follows:

1. Lichen is not a disease, but a type of lesion.

2. The term should be reserved for the clinical entity, described by Erasmus Wilson under the name of lichen planus, which is the same as Hebra's lichen ruber.

3. The affection described by Kaposi under the name of lichen ruber acuminatus, is identical with that described by Devergie & Bessnier as pityriasis rubra pilaris.

4. Other forms of lichen-obtusus, hypertrophicus, verrucosus, etc., are variants of the typical form, the Hebra-Wilson lichen ruber planus.

5. The groups of symptoms to which the name of lichen is applied is probably caused by a variety of factors, but at present we are almost entirely in the dark as to its pathogenesis. (Malcolm Morris, M. D., British Journal of Dermatology, April, 1894.)

---

HYDROA VACCINIFORME OF BAZIN, (C. Boeck in *Norsk Magazin* for Laegevidenskaben, No. 6, 1893).—Three cases of this rare affection are reported. All bears out the classical description of the disease. The report is especially interesting in that the third case instanced occurred in a young woman of twenty-seven years.

(Although Bazin was the first to describe this condition, Hutchinson probably deserves the credit of making it recognizable. Crocker, as late as 1893, describes the disease as one of early childhood and confined to boys. Hutchinson designated the disease as "Recurrent Summer Eruption," while Unna named it "Hydroa Buerorum," believing it a disease of boys. The disease is one of the symmetrical vesicular eruptions, suggesting a vaso-motor neurosis. In Bazin's case, vaccination seemed responsible.—Dyer.)

In all three of Boeck's cases the lesions terminated in marked cicatrization. (Ibid.)

---

MEDICATED BATHS IN THE TREATMENT OF SKIN DISEASES, by Leslie Phillips, M. D.; H. K. Lewis, London, 1893.—So little is known of the usefulness of baths in the treatment of skin diseases, that Dr. Phillips' contribution is most welcome. The brevity of the work is its chief fault. The author devotes considerable space to the consideration of the ordinary indications for the bath, the uses of water alone and the relation of bathing to the general functions of the economy. After describing in detail the various baths and their composition, the author indicates the conditions of which these are separately indicated. In commending the entire work, special mention should be made to Part III, in which the application of specific baths to specific diseases is given. The usually accepted dictum that water is contra-indicated in eczemas, finds refutation here.

Says Dr. Phillips, "Since I have learned to give baths of tepid temperature only, to cases of acute eczema, I have constantly been on the lookout for a case in which the bath injures or fails to benefit, and I have not yet found it." This is rather strongly put, but the accompanying description of methods in employing the bath in eczema somewhat qualifies the above. In commenting on this position we would say that the tepid or even hot bath in our experience has proven of service in selected cases. A prolonged bath, even to exhaustion, from half an hour to two hours or more should be the rule, however.

Medication through the bath is gaining ground and in submitting this humble effort, the doctor has done much in the line of enlightenment in this direction.

---

TRICHOTILLOMANIA.—At the May meeting of the Paris Society of Dermatology and Syphilography, Dr. Hallopeau reported a case of this remarkabe condition. The patient was reported in a morbid state, characterized by violent itching in the hairy regeons of the body, which forced him to seek relief by tearing out the hair of the part affected. Careful examination discovered no alteration of the hair or integument. Relief was only obtained by protecting the itching parts with a varnish or India-rubber covering.—*Medical Weekly*, v. ii., No. 21, p. 248.

